# Ocular Filariasis caused by *Loa loa* infection

**DOI:** 10.1590/0037-8682-0044-2025

**Published:** 2025-06-02

**Authors:** Leonor Murça da Silva Balo, Howard Lopes Ribeiro

**Affiliations:** 1 Principal Military Hospital / Higher Institute, Luanda, Angola.; 2 Núcleo de Pesquisa e Desenvolvimento de Medicamentos (NPDM), Universidade Federal do Ceará, Fortaleza, CE, Brasil.

**FIGURE 1:**
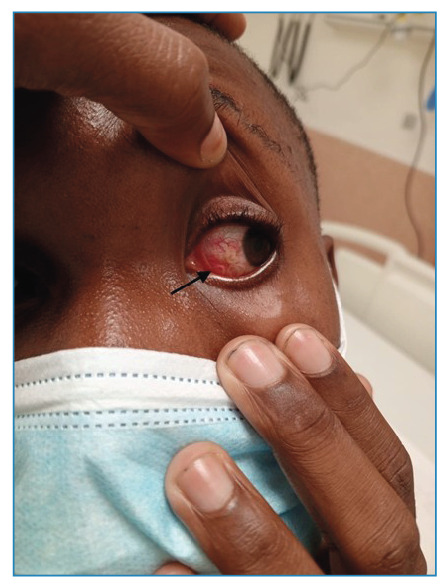
Ocular loiasis. Visible signs of infection, including conjunctival erythema and the presence of the parasite.

**FIGURE 2:**
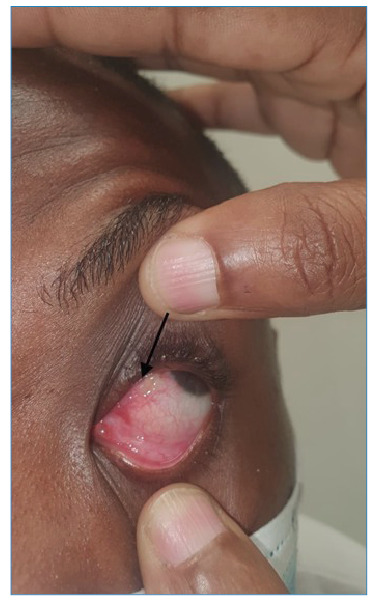
Observation of a *Loa loa* parasite beneath the conjunctive.

A 27-year-old soldier stationed in Cabinda, Angola, reported four days of left-sided eye itching and a sensation of a foreign body. Despite the absence of an ophthalmologist, ocular loiasis was diagnosed, and he was evacuated to the central military hospital in Luanda. His symptoms intensified, manifesting as increased eye discomfort and blurred vision. Examination revealed ocular pain, redness, light sensitivity, and a mobile parasite under the conjunctiva, confirming a Loa loa infection (Figures 1 and 2, and clinical video). Loiasis, endemic to Central and West Africa, is transmitted by the Chrysops fly, with mature worms traveling beneath the skin and occasionally migrating into the eye. This can lead to symptoms such as Calabar swellings, itching, and eye discomfort. These parasites can persist for years, causing recurrent symptoms that affect quality of life.

The diagnosis relies on clinical signs, coupled with the identification of parasites, eosinophilia, and microfilariae in blood smears^1^ ^,^ ^2^. In this case, treatment involved 1% prednisolone eye drops administered four times daily for one week, diethylcarbamazine at a dosage of 6 mg/kg/day for 21 days, and a single dose of ivermectin ranging from 150-200 µg/kg. Surgical intervention was unnecessary as the worm exited spontaneously. In cases with significant microfilaremia, careful monitoring is essential due to the potential for severe reactions, such as encephalopathy^3^. At a one-week follow-up, the patient exhibited no symptoms and no further ocular parasites were detected. Ocular loiasis remains a critical differential diagnosis in endemic areas, necessitating prompt recognition and treatment to prevent complications such as vision loss, secondary infections, or progression to systemic involvement^4^ ^,^ ^5^. This case underscores the need for heightened clinical vigilance and early intervention to mitigate ocular and systemic complications associated with Loa loa infection, including keratitis, uveitis, encephalopathy, and potential renal or cardiac involvement.
